# What Israeli policy can teach us about elective sex selection

**DOI:** 10.1186/2045-4015-3-42

**Published:** 2014-12-18

**Authors:** Gila Leiter

**Affiliations:** Department of Obstetrics, Gynecology, and Reproductive Science, Icahn School of Medicine at Mount Sinai, Mount Sinai Health System, New York, NY USA

## Abstract

PIGD for gender selection for non medical reasons has been a subject of ethical, legal, and moral debate in many Western countries.

This article discusses the background of elective sex selection, and highlights the impact of new technological developments on this dynamic discussion. The article published by Pessach et al., in this Journal, is an excellent study of Israeli health policy on non medically indicated preimplantation genetic screening for sex selection.

In Israel, elective sex selection is prohibited, but exceptions can be made by application, for family balancing, and emotional and religious reasons. This review of a health policy over seven years is concordant with evolving views in many Western countries. The classic medical model for allowing sex selection for serious medical disorders may be too restrictive. There are different reasons that may be assessed in light of ethical criteria including a wider delineation of medical reasons, which may include emotional and psychological well being of the family, indirect medical reasons, as well as risk reduction for the following generations.

The Israeli model may be a useful approach with wide application to reproductive health policies in many countries.

Sex selection is a complex and charged issue and new technological developments make the discussion more urgent. The article by Pessach et al. [1] provides a review and careful analysis of the novel approach in Israel to a controversial ethical and social issue, the use of Preimplantation Genetic Diagnosis for *elective* gender selection.

Sex selection can be performed *preconception*-by sperm selection, *preimplantation* -using embryo screening, and *prenatally*, using sex selective abortion. Technological advances in each of these areas has increased availability for sex selection, making this a subject of recurrent ethical, legal ,and health policy debates in many countries.

These methods vary in their reliability, risks, costs, and moral acceptability.

Globally, sex selection has serious societal impacts as it has been used mostly to abort females and has impact on population sex ratios, with far reaching consequences [2]. This trend in countries such as India and China has been reversing, and to date in Western countries distortion of the sex ratio has not been a great concern, and preference for sons is weak or absent [3–5]. However, with the increasing trend to perform genetic screening of embryos before implantation in IVF cycles, to avoid aneuploidy and improve outcomes, as well as reliable non invasive fetal testing (NIFT) very early in pregnancy, (both of which reveals embryo sex), it remains to be seen if a distorted sex ratio will develop in Western countries in the future.

Sex selection for medical reasons is accepted by most countries, and legislation usually defines this as gender selection to prevent serious sex related medical diseases, such as Duchenne Muscular Dystrophy, or hemophilia.

In Israel, sex selection for medical reasons is included in the basic benefits package to which all Israelis are entitled, within the framework of National Health Insurance. In addition, hospital-based pregnancy termination committees in Israel are authorized to approve abortions related to sex selection for medical reasons.

Other countries allow sex selection to prevent diseases with unequal sex incidence and severity, such as Fragile X Syndrome and autism. Other medical concerns such as decreasing transmission of disease to a third generation, thereby avoiding a high genetic risk for future healthy children, are currently being debated [6, 7]. An example of this is a male patient with an x linked recessive disease like hemophilia, who prefers to have sons only, so that his daughters will not be obligate carriers, with a 25% risk of transmitting the disease. This attempt to improve trans-generational health may be considered an indirect medical reason. Another example is that in order to prevent passing on a woman’s mitochondrial DNA disorder with the concomitant possibility of increasing her mutation load in future generations, parents may want to select only for boys. This is an area of debate, and legislation in countries like Germany and England do not clearly spell this out [8]. Most legislation and jurisdiction applies only to using techniques of Assisted Reproductive Technology for sex selection, whereas professional regulation and guidelines are used to advise against sex selective abortion.

The ethics of gender selection for non medical reasons touches on many important areas. Concerns about human dignity and respect for all persons, and the reinforcement of gender role expectations and sexist motives inform the debate. Gender selection invariably leads to discussion about the limits of procreative liberty, and the “slippery slope” leading to designer babies, where Preimplantation Genetic Diagnosis may be used to select traits like eye color or height. Other issues raised are about the health of the offspring chosen by sex selection and whether undue expectations of such children may be harmful. The most effective sex selection is through PGD in IVF cycles, where embryo biopsy is done at the cleavage or blastocyst stage. IVF confers (small) risks to mother, hefty costs, and embryos which will be created and then destroyed only because of their sex, clearly disturbing to those who believe, as the ESHRE Task Force on Ethics and Law stated “the embryo is owed respect as a symbol of future human life” [9].

Opponents to limiting gender selection through PGD consider concerns about sexist discrimination or entrenching patriarchal values as being largely symbolic, and limiting sex selection infringes on reproductive freedom and self determination. Evidence for distorted sex ratios in Western countries, or harm to child selected is lacking, and thus gender selection through PGD should be allowed.

Many families request sex selection for family balancing, selecting an embryo that is the opposite sex of one or more of their existing children. The goal is for gender variety in a family, and is frequently motivated by the female partner, and thus is not inherently sexist, as it may be motivated by the desire to rear children of both genders.

In US there is no legislation preventing PGD for sex selection, despite guidelines against it, and it is apparently being performed upon parental request at many clinics.

In 36 countries there is legislation against sex selection using ART, including 25 European countries. ESHRE (European society of human reproduction and embryology) recently revisited the debate about social sexing [8], and divergent views about the current ban were described, which may allow for reevaluation of the current legislation in these countries. This may allow greater use of sex selection in various circumstances that take account of societal concerns about the impact of its practice.

Israel has a unique approach to the question. Israel is known for its high rates of IVF with the highest rates of intervention per capita in the world [10]. PGD is regulated by the Ministry of Health, since 2005, based not on legislation, but mandated by directive, and sex selection for non medical reasons is basically prohibited. Exceptions can be made in rare cases, after written permission is granted by the committee, appointed by the director general of the Ministry of Health and composed of seven members, including a psychologist, physicians in Ob/Gyn AND genetics, a medical ethicist, social worker, lawyer, and clergyman.

Couples and single women can apply if they feel there would be significant damage to the family member’s mental health if the procedure was not done. The applicants should be married and have four joint children of same sex and none of other. They must undergo genetic counseling about the PGD process, and give written consent. The applicants must understand if embryos of the non-selected sex remain, no additional IVF cycles can be done until all remaining embryos are used by the couple. In addition, specific and “idiosyncratic” religious reasons were considered, as in cases of priestly Jewish families, requiring a sperm donor, where a son who is not of genetic lineage could not publicly bless the congregation, and thus girls would be preferred [11].

Also included in the application was whether IVF was necessary to achieve a pregnancy in any event. The procedure was not funded by the government, unlike the liberal IVF coverage for infertility in Israel.

Pessach et al. reviewed all the 411 applications received during the first seven years of the committee’s activities.

Their careful analysis included demographics, reasons for requesting PGD sex selection, fertility treatments, preferred sex, and psychological assessment once application was considered, in order to assess the risk to the couple if the procedure was denied. Their findings were that a majority of the couples applying and approved met the primary criterion of having four joint children of one sex. Most of the applicants requested male children (100% of Arab families, 2/3 of Jewish applicants), and the primary reason for request was parent’s intense emotional desire. In 78.4% of the applications IVF or other fertility treatments were not necessary to achieve pregnancy.

This unique Israeli policy, which presupposes a general negative attitude towards non medical PGD, attempts to provide a solution in rare cases. There is particular attention to the psychological well being of the family, while minimizing many of the ethical and social concerns of nonmedical PGD.

Family balancing of >4 of one sex, understanding the risks of the procedures, and limiting the number of embryos that may be discarded due to gender, all take into account many of the concerns described above. This limits the number of non medical PGD procedures performed, while still allowing some families meeting criteria to achieve their intense desire.

Many of these principles can be used to resolve this issue in other countries, and adopted to their particular culture.

Psychological and relational factors should be taken into account, as in the Israeli model.

In the Israeli model, greater leniency was shown with couples requiring infertility treatments anyway, and surveys in US have shown that in cases where couples need to undergo PGD or IVF anyway, more will request PGD for sex selection. The criteria of proportionality seems to be met more readily in the cases where IVF or ICSI needs to be performed for infertility [6]. This is not universal, as in Germany 90% of respondents in surveys, would not use PGD for sex selection for non medical reasons.

As preimplantation genetic screening in IVF for infertility becomes more routine, and the sex of fetus may be known, it will be harder to restrict non-medical sex selection and important to study its impact. Similarly, with the availability of NIFT done early in gestation, which is less regulated, it will be imperative to monitor its effect on sex ratios in all countries.

The authors are to be lauded for a careful and insightful analysis of an approach to non-medical sex selection by PGD, which can serve as a template for others.

## Author information

Dr. Gila Leiter is an Associate Clinical Professor at Icahn School of Medicine at Mount Sinai, Department of Obstetrics, Gynecology, and Reproductive Sciences in New York.

She is an attending physician at Mount Sinai Hospital, and a member of the Administrative Executive Committee, and Secretary of the Medical Board at Mount Sinai Hospital. She has lectured extensively on issues of reproductive health ethics.

## Commentary on

Pessach, N., Glasser, S., Soskolne, V., Barash, A. & Lerner-Geva, L. The Israeli National Committee for sex selection by pre-implantation genetic diagnosis: a novel approach (2005-2011). *Israel journal of health policy research***3**, 33, doi:10.1186/2045-4015-3-33 (2014).
